# Kidney phosphate wasting predicts poor outcome in polycystic kidney disease

**DOI:** 10.1093/ndt/gfad247

**Published:** 2023-11-20

**Authors:** Laixi Xue, Frank Geurts, Esther Meijer, Martin H de Borst, Ron T Gansevoort, Robert Zietse, Ewout J Hoorn, Mahdi Salih, the DIPAK Consortium, Joost P H Drenth, Johannes W de Fijter, Monique Losekoot, Dorien J M Peters, Jack F Wetzels, Tom Nijenhuis

**Affiliations:** Department of Internal Medicine, Division of Nephrology and Transplantation, Erasmus Medical Center, Rotterdam, The Netherlands; Department of Internal Medicine, Division of Nephrology and Transplantation, Erasmus Medical Center, Rotterdam, The Netherlands; Department of Nephrology, University Medical Center Groningen, Groningen, The Netherlands; Department of Nephrology, University Medical Center Groningen, Groningen, The Netherlands; Department of Nephrology, University Medical Center Groningen, Groningen, The Netherlands; Department of Internal Medicine, Division of Nephrology and Transplantation, Erasmus Medical Center, Rotterdam, The Netherlands; Department of Internal Medicine, Division of Nephrology and Transplantation, Erasmus Medical Center, Rotterdam, The Netherlands; Department of Internal Medicine, Division of Nephrology and Transplantation, Erasmus Medical Center, Rotterdam, The Netherlands

**Keywords:** ADPKD, FGF-23, kidney failure, phosphate, TmP/GFR

## Abstract

**Background:**

Patients with autosomal dominant polycystic kidney disease (ADPKD) have disproportionately high levels of fibroblast growth factor 23 (FGF-23) for their chronic kidney disease stage, however only a subgroup develops kidney phosphate wasting. We assessed factors associated with phosphate wasting and hypothesize that it identifies patients with more severe disease and predicts disease progression.

**Methods:**

We included 604 patients with ADPKD from a multicenter prospective observational cohort (DIPAK; Developing Intervention Strategies to Halt Progression of Autosomal Dominant Polycystic Kidney Disease) in four university medical centers in the Netherlands. We measured parathyroid hormone (PTH) and total plasma FGF-23 levels, and calculated the ratio of tubular maximum reabsorption rate of phosphate to glomerular filtration rate (TmP/GFR) with <0.8 mmol/L defined as kidney phosphate wasting. We analysed the association of TmP/GFR with estimated GFR (eGFR) decline over time and the risk for a composite kidney outcome (≥30% eGFR decline, kidney failure or kidney replacement therapy).

**Results:**

In our cohort (age 48 ± 12 years, 39% male, eGFR 63 ± 28 mL/min/1.73 m^2^), 59% of patients had phosphate wasting. Male sex [coefficient –0.2, 95% confidence interval (CI) –0.2; –0.1], eGFR (0.002, 95% CI 0.001; 0.004), FGF-23 (0.1, 95% CI 0.03; 0.2), PTH (–0.2, 95% CI –0.3; –0.06) and copeptin (–0.08, 95% CI –0.1; –0.08) were associated with TmP/GFR. Corrected for PTH, FGF-23 and eGFR, every 0.1 mmol/L decrease in TmP/GFR was associated with a greater eGFR decline of 0.2 mL/min/1.73 m^2^/year (95% CI 0.01; 0.3) and an increased hazard ratio of 1.09 (95% CI 1.01; 1.18) of the composite kidney outcome.

**Conclusion:**

Our study shows that in patients with ADPKD, phosphate wasting is prevalent and associated with more rapid disease progression. Phosphate wasting may be a consequence of early proximal tubular dysfunction and insufficient suppression of PTH.

## Graphical Abstract

**Graphical Abstract:**
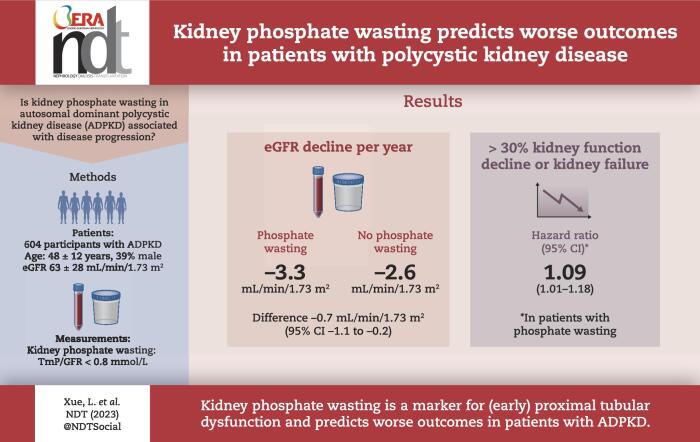

KEY LEARNING POINTS **What was known:**

- The phosphaturic hormone fibroblast growth factor 23 (FGF-23) is elevated in patients with autosomal dominant polycystic kidney disease (ADPKD) compared with healthy individuals, and patients with non-ADPKD chronic kidney disease.
- Higher FGF-23 levels are associated with disease progression in ADPKD.
- Despite high FGF-23 levels, only a subgroup of patients with ADPKD develops kidney phosphate wasting.

**This study adds:**

- This study directly compared patients with and without phosphate wasting.
- Kidney phosphate wasting is common (59%) in patients with ADPKD, and is associated with higher parathyroid hormone (PTH) levels, but independent of FGF-23 levels.
- Kidney phosphate wasting is associated with disease progression in ADPKD independent of PTH, FGF-23 and other known predictors of disease progression.

**Potential impact:**

- Kidney phosphate wasting may be caused by early proximal tubular dysfunction and inadequately high PTH levels and may serve as an additional prognostic factor in ADPKD.
- Kidney phosphate wasting may contribute to disease progression in ADPKD through mechanisms independent of FGF-23.

## INTRODUCTION

Autosomal dominant polycystic kidney disease (ADPKD) is the most common inherited kidney disease and is characterized by the development and expansion of bilateral cysts often leading to kidney failure [1]. In patients with ADPKD, levels of the phosphaturic hormone fibroblast growth factor 23 (FGF-23) are 2- to 3-fold higher compared with those with non-ADPKD chronic kidney disease (CKD) and 6-fold higher compared with healthy subjects despite similar levels of parathyroid hormone (PTH), vitamin D and serum calcium [2–5]. The reason for the disproportionately higher FGF-23 levels in ADPKD is unclear, but may be caused by increased secretion by osteocytes or ectopic production by kidney or hepatic cysts [2, 3, 6]. Two observational studies reported that a higher FGF-23 level is an independent risk factor for disease progression in ADPKD [7, 8].

Despite high levels of FGF-23, only a subgroup of patients with ADPKD and CKD stages G1 and G2 develop kidney phosphate wasting, defined as a maximal tubular reabsorption ratio of phosphate to glomerular filtration ratio (TmP/GFR) of <0.8 mmol/L, with or without hypophosphatemia [4, 5]. This discrepancy suggests that FGF-23 may not be the only driver of phosphate wasting in patients with ADPKD. Patients with or without phosphate wasting have not been directly compared with each other.

Therefore, in the present study, we characterized patients with ADPKD with or without kidney phosphate wasting in a large prospective observational cohort. Additionally, we assessed whether kidney phosphate wasting is associated with annual estimated GFR (eGFR) decline rate and a composite kidney outcome defined as ≥30% eGFR decline, the development of kidney failure or initiation with kidney replacement therapy. Our findings suggest that kidney phosphate wasting is associated with faster eGFR decline and worse kidney survival, and could provide additional predictive value to the traditional risk prediction models.

## MATERIALS AND METHODS

### Study design and participants

All patients from the ongoing prospective multicenter observational cohort DIPAK (Developing Intervention Strategies to Halt Progression of Autosomal Dominant Polycystic Kidney Disease) were eligible for inclusion. In the DIPAK observational cohort, 670 patients with ADPKD were recruited from four university medical centers in the Netherlands (Groningen, Leiden, Nijmegen and Rotterdam) with yearly study visits, including blood and urine collections and investigation of the natural development and disease progression. Patient were included if they were diagnosed with ADPKD based on the modified Ravine criteria [9], aged ≥18 years and had eGFR ≥15 mL/min/1.73 m^2^. The main exclusion criteria for the DIPAK observational cohort were diabetes mellitus and patients with proteinuria >1 g/24 h or use of medication that could influence the natural course of the disease, including the use of nephrotoxic medication such as nonsteroidal anti-inflammatory drugs, lithium and immunosuppressive drugs. For this analysis, we included patients with available baseline plasma and urine phosphate levels. The DIPAK observational cohort was approved by the Institutional Medical Ethical Committee of the University Medical Center Groningen (MEC-2013/040) and conducted in adherence to the International Conference on Harmonization–Good Clinical Practice guidelines and the Declaration of Helsinki. All patients provided written informed consent.

### Measurements

Plasma and urine phosphate, and serum and urine calcium and creatinine levels were measured using routine laboratory procedures on an automated chemistry platform (Cobas 8000, Roche Diagnostics, Mannheim, Germany). We calculated the eGFR using the creatinine-based Chronic Kidney Disease Epidemiology Collaboration formula [10]. Twenty-four-hour urine collections with biologically implausible urinary creatinine excretion (<3.09 or >30.9 mmol/day) were excluded from further analysis [11]. We measured total plasma FGF-23 concentrations using a commercially available enzyme-linked immunosorbent assay (ELISA) (Quidel, San Diego, CA, USA), as performed in previous study in ADPKD [12]. This ELISA kit detects both intact and carboxyl-terminal fragments of FGF-23 and has a detection range up to 1400 RU/mL when read at 620 nm, with an intra- and inter-assay variability coefficient of <5% and <16%, respectively [12]. Serum PTH and 25-hydroxy(OH) vitamin D concentrations were measured using fully automated two-step enzyme immunoassays (Lumipulse G1200 System, Fujirebio Inc., Tokyo, Japan). Urinary albumin was measured with a colorimetric assay using bromocresol green (BCG; Sigma Aldrich Co. LLC, St Louis, MO, USA). Plasma copeptin was measured using a sandwich immunoassay (BRAHMS, Thermo Fisher Scientific, Berlin, Germany). β2-microglobulin was measured by ELISA assay (Anogen-Yes Biotech Laboratories Ltd, Mississauga, Canada). The lower-level of detection was 18 ng/mL, and the intra- and inter-assay variation was 6.3% and 8.5%, respectively. Monocyte Chemoattractant Protein-1 (MCP-1) was measured by ELISA (R&D Systems Inc., Minneapolis, MN, USA). The assay had a lower-level of detection of 0.04 ng/mL, with intra- and inter-assay variation of 8.3% and 12.7%, respectively [13]. Standardized abdominal MRI was performed to assess the total kidney volume (TKV). We estimated TKV on T2-weighted coronal MRI images using the software Analyze direct 9.0 (AnalyzeDirect, Inc., Overland Park, KS, USA) with manual segmentation tracing method and adjusted for height in meters to calculate the height-adjusted TKV (htTKV) [14].

### TmP/GFR calculations

TmP/GFR (mmol/L) was first described by Bijvoet *et al*. [15] and later revised by Payne *et al.* [16] to help differentiate hypercalcemia due to hyperparathyroidism from other causes. It indicates the maximum capacity of the kidney to reabsorb phosphate. A lower TmP/GFR is therefore an indication of kidney phosphate wasting. TmP/GFR is modulated by PTH and FGF-23 such that higher levels of either hormone result in a lower TmP/GFR [17].

First, we calculated fraction excretion of phosphate (FEPi) from 24-h urine samples:


\begin{equation*}FEPi = \frac{{{\mathrm{serum\ creatinine\ \times \ urine\ phosphate\ }}}}{{{\mathrm{urine\ creatinine\ \times \ plasma\ phosphate\ }}}}\end{equation*}

Second, the fractional tubular reabsorption of phosphate (TRP) was calculated by:


\begin{equation*}TRP = 1 - {\mathrm{FEPi}}\end{equation*}

With TRP values ≤0.86, tubular phosphate reabsorption is at its maximum with a linear relationship between plasma phosphate levels and urinary phosphate excretion. TmP/GFR is then calculated by:


\begin{equation*}TmP/GFR\ \left( {mmol/L} \right) = {\mathrm{TRP\ \times \ plasma\ phosphate}}\end{equation*}

With TRP values >0.86, the relationship between plasma phosphate levels and phosphate excretion is curvilinear, and therefore TmP/GFR is calculated by:


\begin{equation*}TmP/GFR\ \left( {mmol/L} \right) = \frac{{0.3{\mathrm{\ \times \ TRP}}}}{{1 - \left( {0.8{\mathrm{\ \times \ TRP}}} \right)}}{\mathrm{\ \times \ plasma\ phosphate}}\end{equation*}

The normal range for TmP/GFR in healthy 25- to 75-year-old individuals is between 0.80 and 1.44 mmol/L [16]. Therefore, a TmP/GFR value <0.8 mmol/L is defined as kidney phosphate wasting.

### Statistical analysis

Continuous data are presented as mean ± standard deviation when normally distributed, or as median ± interquartile range (IQR) when non-normally distributed. Non-normally distributed data were log-transformed prior to analysis when necessary. We divided the cohort into three groups based on their CKD stage at baseline: CKD G1 (eGFR >90 mL/min/1.73 m^2^), CKD G2 (eGFR 60–90 mL/min/1.73 m^2^) and CKD G3/4 (eGFR <60 mL/min/1.73 m^2^). We merged CKD stages G3 and G4 due to limited number of patients with CKD stage G4 (*n* = 65). Group differences were analysed with one-way analysis of variance for continuous variables and Pearson's chi-square test for categorical variables. Four longitudinal analyses were performed. First, we used multivariable linear regression to analyse which baseline variables were associated with TmP/GFR. We included variables that were deemed relevant for assessing kidney outcomes in ADPKD and for phosphate homeostasis/phosphate wasting. These included: baseline demographics [age, sex and body mass index (BMI)]; markers for disease severity (genotype, eGFR, htTKV, plasma copeptin and proteinuria); treatments that may influence disease course (use of tolvaptan and use of diuretics); markers involved in phosphate homeostasis (serum 25-OH vitamin D, serum PTH, serum calcium, total plasma FGF-23 and urine urea excretion). Second, linear mixed effect models were used to assess differences in eGFR slope based on tertiles of TmP/GFR or as a continuous variable. The intercept and slope for every participant was allowed to vary randomly and all other covariates were modeled as fixed effects. Third, we conducted a survival analysis to determine the effect of TmP/GFR on the composite kidney outcome defined as ≥30% eGFR decline, kidney failure (eGFR <15 mL/min/1.73 m^2^) or kidney replacement therapy with Kaplan–Meier survival plots and Cox regression models [8]. To minimize the collinearity between TmP/GFR and plasma phosphate levels in longitudinal analysis, we analysed plasma phosphate as a categorical variable with three levels, including hypophosphatemia (<0.8 mmol/L), normophosphatemia (0.8–1.5 mmol/L, reference) and hyperphosphatemia (>1.5 mmol/L) in the longitudinal analysis. Statistical significance was defined by a two-sided *P*-value of <.05. *P*-values were adjusted for multiple testing with *post hoc* Bonferroni correction, if necessary. Statistical analyses were performed using SPSS software version 29 (IBM, New York, NY, USA).

## RESULTS

### Baseline characteristics

A total of 604 out of 670 participants of the DIPAK observational cohort with available plasma and urine phosphate levels were included in the present analysis (Table 1). Patients were 48 ± 12 years old, 39% were male with an average eGFR of 63 ± 28 mL/min/1.73 m^2^ and an htTKV of 0.9 ± 0.6 L/m. Sixty-one patients were treated with tolvaptan. The median follow-up was 4 years (IQR 2–5 years). The mean TmP/GFR was 0.76 ± 0.23 mmol/L and 357 patients (59%) had kidney phosphate wasting. Hypophosphatemia was present in 49 patients (8%). We subdivided patients into three groups based on their CKD stage. The prevalence of phosphate wasting was higher in more advanced CKD stages: 38%, 54% and 70% for CKD stages G1, G2 and G3/4, respectively (Table 1). Within each CKD stage, patients with kidney phosphate wasting were predominantly males, had a larger htTKV, a lower plasma phosphate concentration, higher PTH and higher plasma copeptin levels (all *P*-values <.05). Notably, their 24-h urinary phosphate excretion was also higher. No differences were observed for Mayo image classification or proteinuria between patients with or without kidney phosphate wasting (data not shown).

**Table 1:** Characteristics associated with kidney phosphate wasting by CKD stages.

		CKD G1			CKD G2			CKD G3/4		
		*n* = 112			*n* = 184			*n* = 308		
CKD stage Kidney phosphate	All	No	Yes		No	Yes		No	Yes	
wasting (TmP/GFR <0.8 mmol/L)	*n* = 604 (%)	*n* = 69 (62)	*n* = 43 (38)	*P*-value	*n* = 85 (46)	*n* = 99 (54)	*P*-value	*n* = 93 (30)	*n* = 215 (70)	*P*-value
Age (years)	48 ± 12	34 ± 11	36 ± 8	.3	46 ± 10	46 ± 9	.6	55 ± 8	53 ± 9	.1
Sex: male, *n* (%)	238 (39)	17 (25)	16 (37)	.1	9 (11)	56 (57)	**<.001**	13 (14)	127 (59)	**<.001**
BMI (kg/m^2^)	26 ± 4	25 ± 5	25 ± 5	.5	26 ± 4	27 ± 4	.9	26 ± 4	27 ± 4	.2
Ethnicity, *n* (%)										
White	577 (96)	65 (94)	40 (93)	1.0	82 (97)	92 (93)	.4	86 (92)	212 (99)	**.01**
Mutation, *n* (%)										
PKD1	399 (67)	53 (79)	32 (74)		53 (64)	58 (60)		63 (68)	140 (65)	
PKD2	143 (24)	9 (13)	9 (21)	.9	23 (28)	25 (26)	.3	19 (20)	58 (27)	.4
htTKV (L/m)	0.9 ± 0.6	0.5 ± 0.2	0.7 ± 0.4	**.002**	0.7 ± 0.4	0.8 ± 0.5	.2	0.9 ± 0.6	1.3 ± 0.8	**.002**
Hypertension, *n* (%)	142 (24)	7 (10)	12 (28)	**.02**	20 (24)	28 (29)	.3	19 (20)	56 (26)	.3
Tolvaptan treatment: yes, *n* (%)	61 (10)	3 (5)	4 (9)	.4	9 (11)	11 (11)	1.0	11 (12)	23 (11)	.8
Diuretics treatment: yes, *n* (%)	124 (21)	4 (6)	6 (14)	.2	10 (12)	12 (12)	1.0	23 (25)	69 (32)	.2
Serum/plasma values										
eGFR (mL/min/1.73 m^2^)	63 ± 28	107 ± 18	103 ± 11	.3	75 ± 9	75 ± 9	.7	41 ± 12	39 ± 11	.2
Calcium (mmol/dL)	2.4 ± 0.1	2.3 ± 0.1	2.3 ± 0.1	.8	2.4 ± 0.1	2.3 ± 0.1	**.005**	2.4 ± 0.1	2.4 ± 0.1	.06
Phosphate (mmol/dL)	1.0 ± 0.2	1.1 ± 0.1	0.9 ± 0.1	**<.001**	1.1 ± 0.1	0.9 ± 0.1	**<.001**	1.2 ± 0.2	1.0 ± 0.1	**<.001**
PTH (pmol/mL)	5.9 ± 3.3	3.6 ± 1.5	4.7 ± 2.0	**.002**	4.5 ± 1.9	5.1 ± 2.3	.06	6.7 ± 3.6	7.9 ± 4.3	**.01**
25-OH vitamin D (nmol/mL)	63 ± 31	57 ± 24	54 ± 24	.6	63 ± 26	52 ± 29	**.02**	71 ± 34	70 ± 33	.8
Copeptin (pmol/L)	8.5 ± 7.4	4.2 ± 2.5	5.4 ± 3.2	**.03**	4.4 ± 2.7	7.1 ± 4.9	**<.001**	9.9 ± 8.3	14.9 ± 11.4	**<.001**
FGF-23 (RU/mL)	121 ± 74	87 ± 51	80 ± 38	.4	100 ± 50	93 ± 47	.3	168 ± 95	155 ± 86	.2
TmP/GFR (mmol/L)	0.76 ± 0.23	1.02 ± 0.17	0.66 ± 0.11	Not tested	0.95 ± 0.13	0.64 ± 0.13	Not tested	0.94 ± 0.13	0.58 ± 0.16	Not tested
Urine excretions										
Calcium (mmol/24h)	2.0 ± 1.7	3.4 ± 2.2	3.6 ± 2.1	.5	2.9 ± 2.0	2.7 ± 1.8	.5	1.8 ± 1.4	1.7 ± 1.2	.4
Phosphate (mmol/24h)	27 ± 10	27 ± 10	30 ± 10	.07	25 ± 9	31 ± 10	**.002**	22 ± 9	28 ± 9	**<.001**
Urea (mmol/24h)	384 ± 128	363 ± 136	393 ± 120	.3	362 ± 125	446 ± 141	**<.001**	338 ± 114	389 ± 116	**<.001**

All continuous variables are expressed as mean ± standard deviation.

Bold values means significant difference with *p* < 0.05.

### Association of TmP/GFR ratio with clinical variables

In our multivariable linear regression model, TmP/GFR was positively correlated with eGFR (β 0.002, 95% CI 0.001; 0.004) and FGF-23 levels (β 0.1, 95% CI 0.03; 0.2), and negatively correlated with male sex (β –0.2, 95% CI –0.2; –0.1), serum PTH (β –0.2, 95% CI –0.3; –0.06) and plasma copeptin levels (β –0.08, 95% CI –0.1; –0.08) (Table 2). No significant correlations were found between TmP/GFR and age, BMI, genotype, htTKV, baseline serum 25-OH vitamin D and serum calcium. Because phosphate accumulation due to CKD inherently results in higher levels of FGF-23 and PTH, we examined the association of TmP/GFR with FGF-23, PTH and calcidiol in patients with relatively preserved CKD stages (G1/2). We found that TmP/GFR is negatively correlated with serum PTH (β –0.3, 95% CI –0.4; –0.1, Supplementary data, Table S1).

**Table 2:** Variables associated with TmP/GFR in a multivariable model.

Baseline values	β (95% CI)	*P*-value
Age, years	0.002 (0.00; 0.004)	.1
Male sex	–0.2 (–0.2; –0.1)	**<.001**
BMI, kg/m^2^	–0.002 (–0.007; 0.002)	.3
*PKD1* genotype	0.01 (–0.03; 0.06)	.6
eGFR, mL/min/1.73 m^2^	0.002 (0.001; 0.004)	**<.001**
htTKV, mL/m	0.05 (–0.04; 0.13)	.3
Tolvaptan treatment	0.02 (–0.05; 0.08)	.6
Diuretic treatment	–0.04 (–0.09; 0.01)	.2
25-OH vitamin D, nmol/mL	–0.01 (–0.1–0.08)	.8
PTH, pmol/mL	–0.2 (–0.3; –0.06)	**.002**
Serum calcium, mmol/dL	0.4 (–0.8; 1.5)	.6
Plasma copeptin, pmol/L	–0.08 (–0.1; –0.08)	**.03**
FGF-23, RU/mL	0.1 (0.03; 0.2)	**.01**
Proteinuria (g/24 h)	0.01 (–0.08; 0.08)	1.0
Urea excretion (mmol/24 h)	–0.00 (–0.00; 0.000)	.3

Log transformation performed in non-normally distributed variables: htTKV, 25-OH vitamin D, PTH, serum calcium, plasma copeptin, FGF-23 and proteinuria.

Bold values means significant difference with *p* < 0.05.

### Association of PTH with plasma phosphate levels

As elevated PTH levels contribute to phosphate wasting, we proceeded to investigate whether PTH was associated with plasma phosphate levels. Our analysis revealed a negative correlation between PTH and plasma phosphate (β –0.1, 95% CI –0.2; –0.1, Supplementary data, Table S2). Additionally, individuals with hypophosphatemia exhibited higher levels of PTH compared with those with normal phosphate levels (5.0 vs 4.3 pmol/mL, *P* = .03, Supplementary data, Table S3).

### Association of TmP/GFR ratio with proximal tubular damage marker and inflammation

Next, we explored the relationship between TmP/GFR and β2-microglobulin and MCP-1, which serve as markers for proximal tubular damage and inflammation, respectively. Our analysis revealed a significant association between TmP/GFR and β2-microglobulin (β –0.06, 95% CI –0.09; –0.03), while a non-significant trend was observed in the association with MCP-1 (β –0.06, 95% CI –0.12; 0.005) (Supplementary data, Table S4).

### Kidney phosphate wasting and eGFR decline

**Figure 1:**
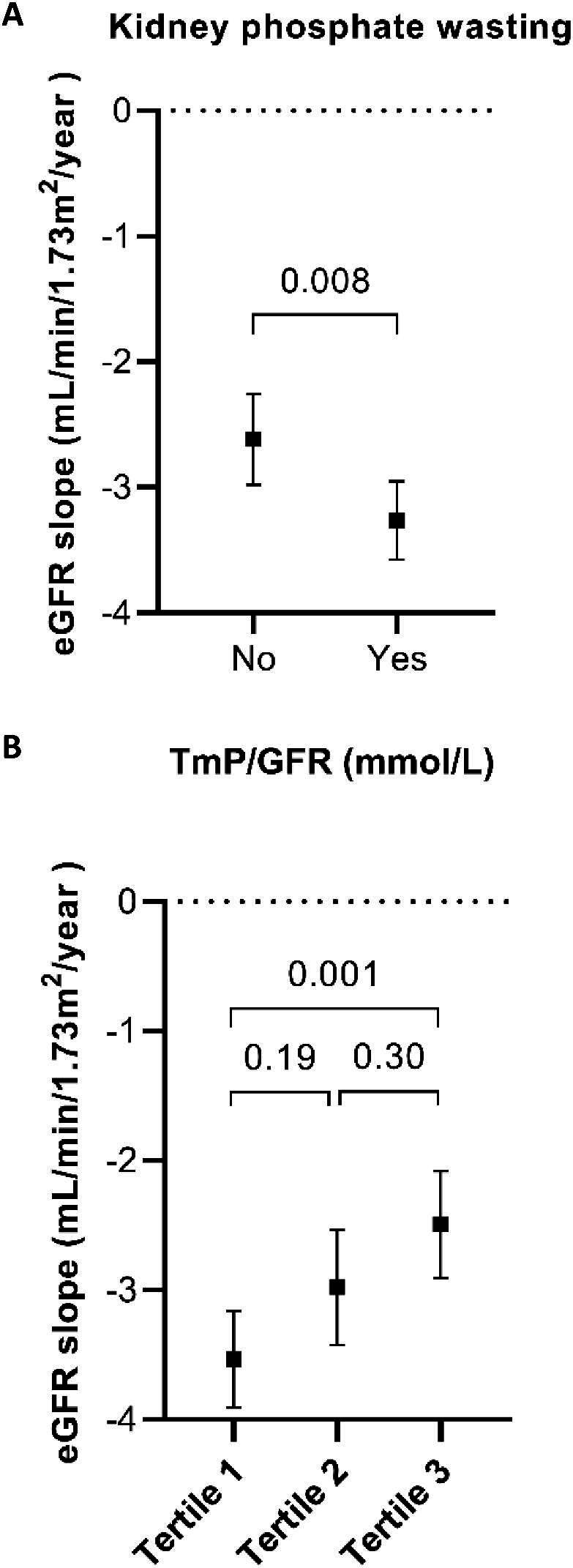
**eGFR slopes according to TmP/GFR.** The plot displays the annual eGFR slopes with black square as mean and corresponding 95% CI in error bars for TmP/GFR by the presence or absence of kidney phosphate wasting (**A**) or divided into tertiles (**B**). Tertile 1: TmP/GFR <0.66 mmol/L; Tertile 2: TmP/GFR 0.66–0.84 mmol/L; Tertile 3: TmP/GFR >0.84 mmol/L.

The average eGFR decline in our cohort was 2.9 mL/min/1.73 m^2^/year. Patients with kidney phosphate wasting had a more rapid eGFR decline than patients without kidney phosphate wasting (3.3 vs 2.6 mL/min/1.73 m^2^/year, *P* = .008, Fig. 1A). When we divided the cohort into tertiles, patients with the most severe phosphate wasting (Tertile 1, TmP/GFR <0.66 mmol/L) had a greater eGFR decline compared with those without phosphate wasting (Tertile 3, TmP/GFR >0.84 mmol/L), 3.5 vs 2.5 mL/min/1.73 m^2^/year (*P* = .001, Fig. 1B). Additionally, we analysed TmP/GFR as a continuous variable in linear-mixed effects models (Table 3). Each 0.1 mmol/L decrease in TmP/GFR was independently associated with a greater eGFR decline of 0.2 mL/min/1.73 m^2^/year (95% CI 0.01–0.3) in the model adjusted for age, sex, BMI, genotype, plasma phosphate, serum PTH, serum 25-OH vitamin D, total plasma FGF-23, plasma copeptin and htTKV.

**Table 3:** Association of TmP/GFR with eGFR slopes.

	TmP/GFR (per 0.1 unit decrease)	TmP/GFR*years (per 0.1 unit decrease)
Unadjusted	–4.1 (–5.0; –3.2)	–0.2 (–0.3; –0.1)
Model 1	–2.4 (–3.2; –1.6)	–0.2 (–0.3; –0.08)
Model 2	–2.8 (–3.6; –2.0)	–0.2 (–0.3; –0.05)
Model 3	–2.3 (–3.1; –1.5)	–0.2 (–0.3; –0.03)
Model 4	–2.2 (–3.0; –1.4)	–0.2 (–0.3; –0.01)

Model 1: adjusted for age, sex, BMI, genotype.

Model 2: Model 1 + tolvaptan treatment, diuretic treatment, plasma phosphate, serum PTH, serum 25-OH vitamin D, total plasma FGF-23.

Model 3: Model 2 + plasma copeptin.

Model 4: Model 3 + htTKV.

### Kidney phosphate wasting and kidney outcomes

**Figure 2:**
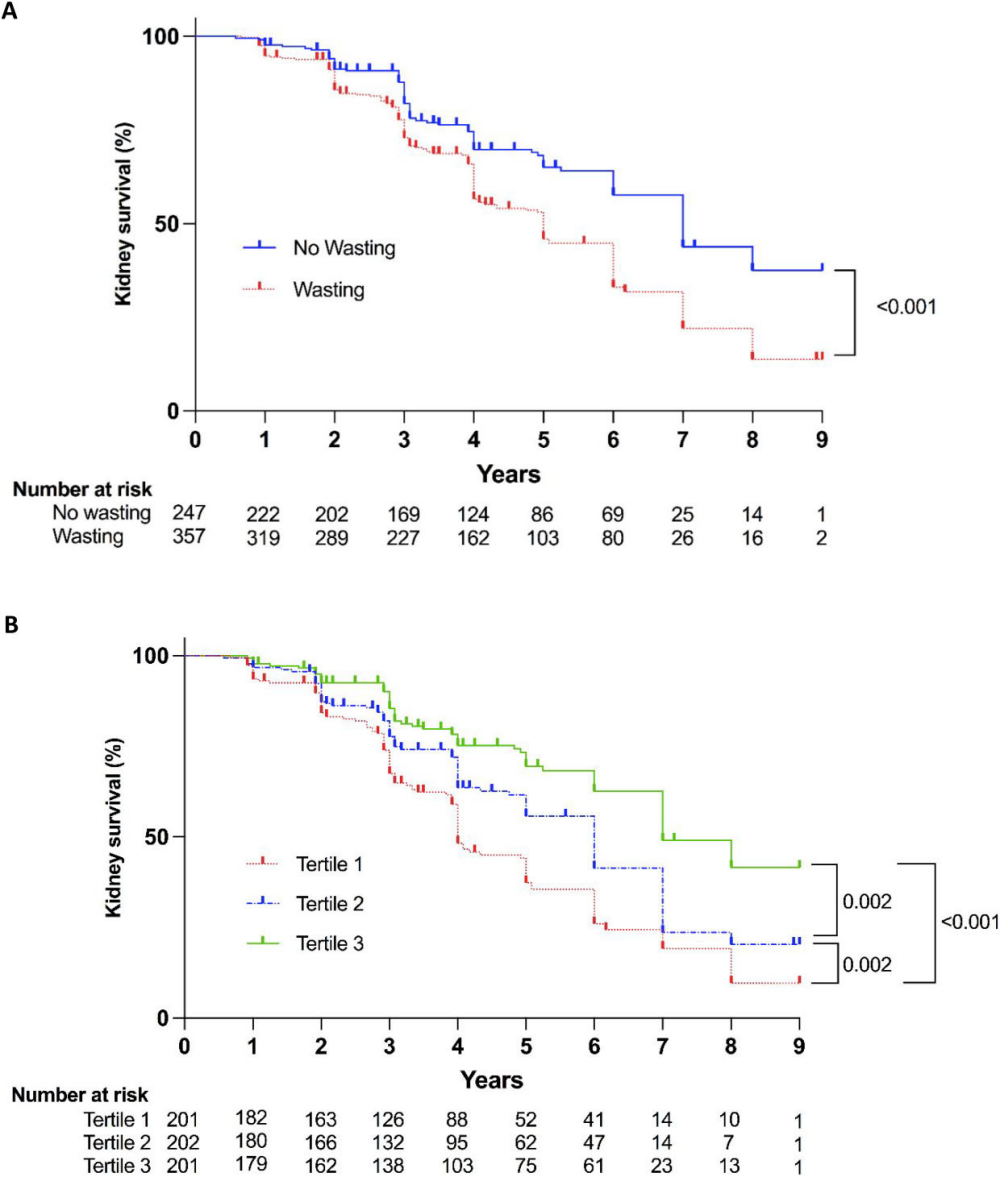
**Kidney survival according to TmP/GFR.** Kaplan–Meier curves for composite kidney outcome stratified by presence or absence of kidney phosphate wasting (**A**) or tertiles of TmP/GFR (**B**). Tertile 1: TmP/GFR <0.66 mmol/L; Tertile 2: TmP/GFR 0.66–0.84 mmol/L; Tertile 3: TmP/GFR >0.84 mmol/L.

**Figure 3:**
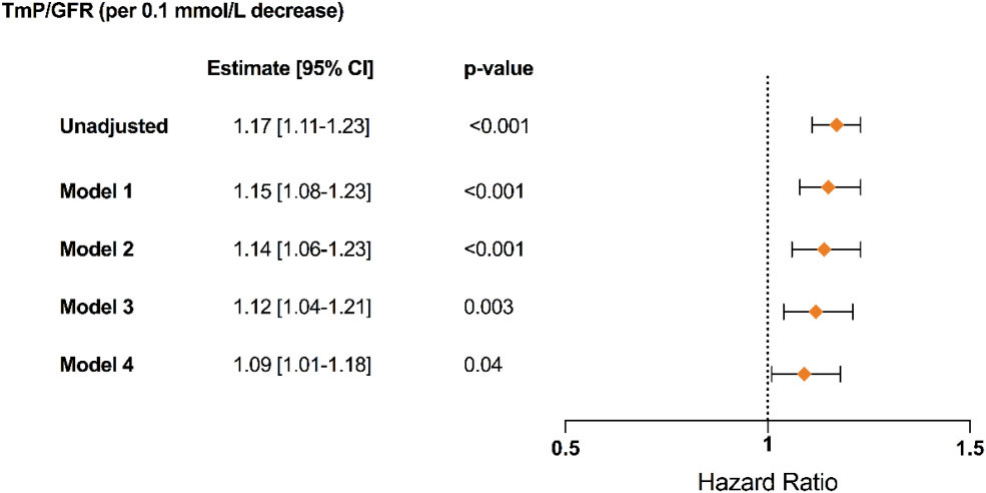
**Kidney outcomes using Cox regression models.** Graphical display of hazard ratios (symbols) with corresponding 95% CI (error bars) for TmP/GFR as continuous variable based on univariate and adjusted Cox regression models (*n* = 604; 271 events). Model 1: adjusted for age, sex, BMI, genotype; Model 2: Model 1 + tolvaptan treatment, diuretics treatment, plasma phosphate, serum PTH, serum 25-OH vitamin D, plasma copeptin, total plasma FGF-23; Model 3: Model 2 + htTKV; Model 4: Model 3 + baseline eGFR.

After a median follow-up of 4 years, we observed 271 composite kidney outcomes: 204 patients had an eGFR decline of ≥30% and 67 patients developed kidney failure. Patients with kidney phosphate wasting were at a significantly greater risk of reaching the composite kidney outcome (Fig. 2A). In addition, those with the most severe phosphate wasting (Tertile 1) had a higher risk for the composite kidney outcome compared with the other two tertiles (Fig. 2B). Next, we assessed the association between TmP/GFR as a continuous variable and the composite kidney outcome in Cox regression models. Every 0.1 mmol/L decrease in TmP/GFR was significantly associated with an increased risk for the composite kidney outcome over time (hazard ratio 1.09, 95% CI 1.01; 1.18), independent of baseline eGFR and other relevant covariates (Fig. 3).

## DISCUSSION

**Figure 4:**
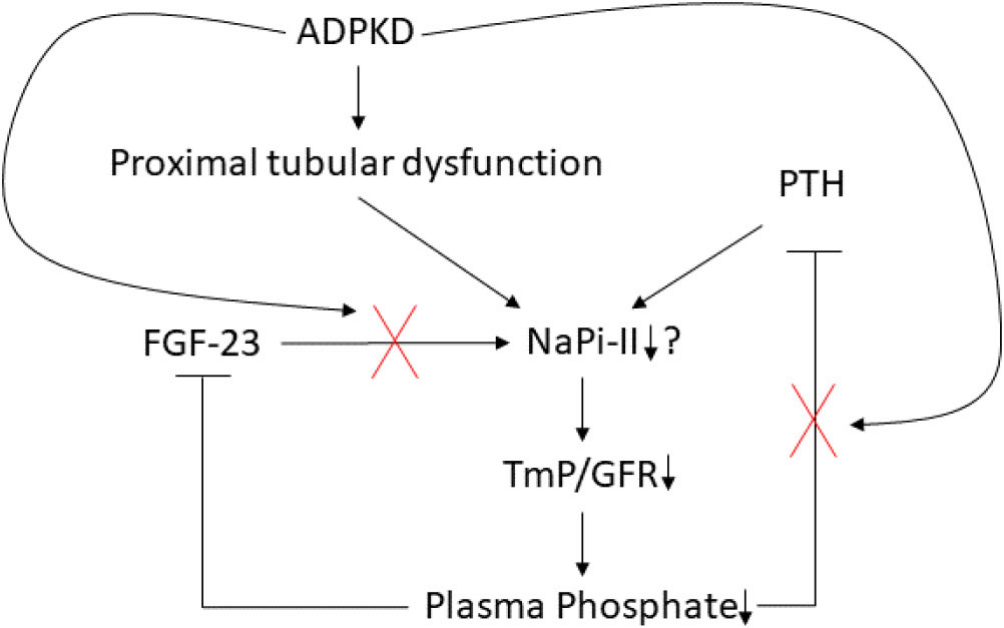
**Proposed hypothesis for the mechanisms of kidney phosphate wasting in ADPKD.** In the context of ADPKD, phosphate wasting may be influenced by both proximal tubular dysfunction and insufficient suppression of PTH. On one hand, the formation and expansion of cysts can disrupt the intricate kidney architecture, resulting in early dysfunction of the proximal tubules. Consequently and potentially, the expression of the sodium phosphate cotransporter NaPi-II diminishes, leading to hypophosphatemia. This, in turn, should prompt the suppression of PTH, but paradoxically, it remains inadequately suppressed, exacerbating the loss of phosphate. Furthermore, the kidney exhibits insensitivity to the effects of total plasma FGF-23.

In this study, we show that kidney phosphate wasting is highly prevalent in patients with ADPKD and occurs more frequently in males with higher levels of copeptin, both established risk factors for rapid disease progression [18–21]. In addition, kidney phosphate wasting is associated with worse kidney outcomes, including a greater annual eGFR decline and a higher risk of the composite kidney outcome (≥30% eGFR decline, kidney failure or kidney replacement therapy). This association was independent of other known risk factors for disease progression and independent of PTH and FGF-23. Below, we will discuss the role of PTH and FGF-23 in kidney phosphate wasting and the question of whether kidney phosphate wasting in ADPKD is a reflection of disease severity or contributes to disease progression (Fig. 4).

The physiological regulators of kidney phosphate reabsorption include serum phosphate concentration, PTH and phosphatonins, with FGF-23 being the most important one.

In response to mild phosphate depletion, there is an increase in the synthesis of the type II sodium/phosphatecotransporter (NaPi-II), which, in turn, stimulates phosphate reabsorption [22]. Intriguingly, our study revealed that individuals with low serum phosphate levels exhibited increased phosphate wasting. These findings are in line with the concept that reduced phosphate levels result from phosphate wasting itself and suggest a potential malfunction in this feedback mechanism, which may involve proximal tubular damage, PTH or FGF-23. Of interest, individuals experiencing phosphate wasting demonstrated elevated 24-h urine phosphate excretion.

Moreover, PTH levels were significantly elevated in those with phosphate wasting, and this elevation persisted even among individuals with hypophosphatemia. Consequently, the insufficient suppression of PTH may contribute to phosphate wasting within our study (Fig. 4). However, it is crucial to note that PTH levels remained within the normal range, and we did not observe any differences in calcium levels that may explain the elevated PTH levels. To the best of our knowledge, only one other study has delved into this aspect in more detail, which did not find a significant difference in PTH between ADPKD patients with or without phosphate wasting at early CKD stages (G1/2), although their cohort was smaller (*n* = 13 vs *n* = 86, with and without phosphate wasting) [4]. Our results further suggest that FGF-23 is not the primary factor driving phosphate wasting in ADPKD as FGF-23 levels were similar in individuals with and without phosphate wasting within each CKD stage. Additionally, in our multivariable analysis, higher total plasma FGF-23 levels were associated with a higher TmP/GFR (indicating no phosphate wasting). A possible explanation for this positive association is a differential expression of membrane-bound Klotho, a co-receptor that is necessary to activate the FGF-23 receptor (FGFR1c), and its downstream signaling [23]. In the presence of Klotho, the FGFR1c downregulates the membrane expression of NaPi-II in the proximal tubule, which leads to phosphate wasting. Conversely, in the absence of Klotho, kidney phosphate wasting is prevented. A previous study found that patients with ADPKD without phosphate wasting had lower levels of soluble Klotho [4]. Notably, in one study utilizing the Han:SPRD (cy/+) rat model and an inducible PKD1 animal model of polycystic kidney disease (PKD), FGF-23 levels were increased, while tissue levels of Klotho did not exhibit any significant differences. Intriguingly, these animals (similar to humans) did not display phosphate wasting [6]. It remains unclear whether soluble Klotho levels accurately reflect membrane-bound Klotho expression in the kidney. In addition, commercially available ELISAs have shown poor inter- and intra-assay agreement [24].

In ADPKD, kidney phosphate wasting may be a direct consequence of the disease, since kidney phosphate wasting may be considered a form of proximal tubular dysfunction [25]. Already during the early stages of ADPKD, cyst formation disrupts the kidney architecture, with the proximal tubule being more prone to cystogenesis and therefore more vulnerable to early damage [26–28]. For example, in another study that used the Han:SPRD (cy/+) rat model of PKD, proximal tubular expression of NaPi-II is reduced during early stages of the disease, resulting in phosphate wasting [28]. This is in line with other early proximal tubular defects, including disturbed ammoniagenesis, impaired proximal tubular secretion and increased urinary excretion of kidney injury molecule 1 and β2-microglobulin [13, 29–32]. In our current study, we observed a significant association between TmP/GFR and β2-microglobulin, providing further evidence that kidney phosphate wasting reflects tubular injury. Taken together, kidney phosphate wasting, defined by a low TmP/GFR, may therefore be regarded as an early marker of proximal tubular dysfunction, and thereby associated with long-term kidney outcomes.

Kidney phosphate wasting may also contribute to cyst formation and disease progression. In the Han:SPRD (cy/+) rat model of PKD, lodged calcium-phosphate microcrystals induce tubule dilation and cyst formation through the activation of the mammalian target of rapamycin (mTOR) and signal transducer and activator of transcription 3 (STAT3) [27]. Treatment with the mTOR inhibitor rapamycin or with potassium citrate diminished these effects. Additionally, calcium-phosphate microcrystals may contribute to inflammation, fibrosis and kidney function through activation of the Toll-like receptor 4 in proximal tubular cells [33]. Phosphate wasting may cause higher supersaturation levels of the calcium-phosphate product in the proximal tubule and thereby promote microcrystal formation, inflammation and fibrosis. In line with this, we recently observed that lower urinary citrate excretion is associated with disease progression in early ADPKD, possibly through similar mechanisms [34].

In this study, and to the best of our knowledge for the first time, we observed that kidney phosphate wasting in patients with ADPKD is an independent risk factor for disease progression. The main strength of our study is the large number of participants with ADPKD, with extensive phenotyping, genotyping and standardized follow-up visits. There are also a number of limitations in our study that should be mentioned. First, we measured urine phosphate excretion and calculated the TmP/GFR ratio based on 24-h urine samples. We recognize that dietary intake can impact 24-h urine phosphate excretion. However, we contend that individuals with phosphate wasting exhibit lower serum phosphate levels compared with those without, supporting the notion of genuine phosphate wasting. Second, although hyperphosphatemia is associated with increased cardiovascular risk in CKD, our study indicates that kidney phosphate wasting, leading to reduced plasma phosphate levels, is associated with an elevated risk of CKD progression, which is an independent risk factor for cardiovascular disease. Further research is warranted to explore this relationship. Third, we have not measured plasma calcitriol (1,25-dihydroxy vitamin D) levels in this study, but we acknowledge its potential value in exploring the underlying mechanism of phosphate wasting. Fourth, it is worth noting that FGF-23 exhibits relatively high variability compared with other parameters, such as PTH and plasma phosphate, which could pose challenges in terms of statistical analysis. Additionally, we measured total FGF-23. Consequently, we cannot differentiate between the measured quantities of C-terminal and intact FGF-23. It is crucial to note that only intact FGF-23 could activate FGFR1c and reduce NaPi-II abundance in the proximal tubule [35]. Another limitation is the predominance of white patients in our study, which may restrict the generalizability of our findings to people with different ethnic backgrounds. Finally, our study has limited generalizability beyond ADPKD because we did not compare our findings with a control group (i.e. patients with non-ADPKD CKD). Consequently, it is unclear whether TmP/GFR is a biomarker for disease progression solely in ADPKD or if it is also relevant in other types of CKD.

In conclusion, our study suggests that a low TmP/GFR is a marker for early proximal tubular dysfunction and therefore predicts worse outcomes in patients with ADPKD.

## Supplementary Material

gfad247_Supplemental_File

## Data Availability

The data underlying this article will be shared on reasonable request to the corresponding author.

## Notes

The collaborators of the DIPAK Consortium are listed in the Acknowledgements.
