# Supplementary material for: Kidney phosphate wasting predicts poor outcome in polycystic kidney disease
Source: Nephrol Dial Transplant. 2023 Nov 20;39(7):1105–14. doi: 10.1093/ndt/gfad247 (PMC11249971; doi:10.1093/ndt/gfad247)
Supplement: gfad247_Supplemental_File [file gfad247_Supplemental_File.docx]

**SUPPLEMENTARY MATERIAL**

**Table S1. Variables associated with TmP/GFR in a multivariable model**

|  | **Beta [95% CI]** | **p-value** |
| --- | --- | --- |
| PTH (pmol/mL) | -0.3 [-0.4; -0.1] | **<0.001** |
| FGF-23 (RU/mL) | 0.04 [-0.07-0.2] | 0.4 |
| 25-OH vitamin D (nmol/mL) | 0.07 [-0.05-0.2] | 0.2 |

***Abbreviations****:* FGF-23: fibroblast growth factor-23; PTH: parathyroid hormone; TmP/GFR: the ratio of tubular maximum reabsorption of phosphate to glomerular filtration rate

All variables were log transformed for analysis

**Table S2. Variables associated with plasma phosphate in a multivariable model**

|  | **Beta [95% CI]** | **P-value** |
| --- | --- | --- |
| PTH (pmol/mL) | -0.11[-0.16;-0.06] | **<0.001** |
| FGF-23 (RU/mL) | 0.044 [-0.001-0.09] | 0.054 |
| 25-OH Vitamin D (nmol/mL) | 0.032 [-0.01-0.07] | 0.1 |

***Abbreviations****:* FGF-23: fibroblast growth factor-23; PTH: parathyroid hormone

All variables were log transformed for analysis

**Table S3. Levels of PTH, FGF-23 and 25-OH vitamin D in patients with or without hypophosphatemia**

|  | **Hypophosphatemia**  **(n= 31)** | **Normophosphatemia**  **(n=256)** | **P-value** |
| --- | --- | --- | --- |
| PTH (pmol/mL) | 5.0 | 4.3 | **0.03** |
| FGF-23 (RU/mL) | 89 | 92 | 0.6 |
| 25-OH Vitamin D (nmol/mL) | 54 | 57 | 0.5 |

***Abbreviations****:* FGF-23: fibroblast growth factor-23; PTH: parathyroid hormone

**Table S4. Markers of tubular damage and inflammation associated with TmP/GFR in univariable model**

|  | **Beta [95% CI]** | **p-value** |
| --- | --- | --- |
| β2-microglobulin, ng/mL | -0.06 [-0.09;-0.03] | **<0.001** |
| MCP-1, pg/mL | -0.06 [-0.12-0.005] | 0.07 |

***Abbreviations****:* MCP-1: monocyte chemoattractant protein 1; TmP/GFR: the ratio of tubular maximum reabsorption of phosphate to glomerular filtration rate

All variables were log transformed for analysis
